# Complete genome sequence of *Hydrogenobacter thermophilus* type strain (TK-6^T^)

**DOI:** 10.4056/sigs.1463589

**Published:** 2011-04-29

**Authors:** Ahmet Zeytun, Johannes Sikorski, Matt Nolan, Alla Lapidus, Susan Lucas, James Han, Hope Tice, Jan-Fang Cheng, Roxanne Tapia, Lynne Goodwin, Sam Pitluck, Konstantinos Liolios, Natalia Ivanova, Konstantinos Mavromatis, Natalia Mikhailova, Galina Ovchinnikova, Amrita Pati, Amy Chen, Krishna Palaniappan, Olivier D. Ngatchou-Djao, Miriam Land, Loren Hauser, Cynthia D. Jeffries, Cliff Han, John C. Detter, Susanne Übler, Manfred Rohde, Brian J. Tindall, Markus Göker, Reinhard Wirth, Tanja Woyke, James Bristow, Jonathan A. Eisen, Victor Markowitz, Philip Hugenholtz, Hans-Peter Klenk, Nikos C. Kyrpides

**Affiliations:** 1 DOE Joint Genome Institute, Walnut Creek, California, USA; 2 DSMZ - German Collection of Microorganisms and Cell Cultures GmbH, Braunschweig, Germany; 3 Los Alamos National Laboratory, Bioscience Division, Los Alamos, New Mexico, USA; 4 Biological Data Management and Technology Center, Lawrence Berkeley National Laboratory, Berkeley, California, USA; 5 HZI – Helmholtz Centre for Infection Research, Braunschweig, Germany; 6 Oak Ridge National Laboratory, Oak Ridge, Tennessee, USA; 7 Archaea Centre - University of Regensburg, Regensburg, Germany; 8 University of California Davis Genome Center, Davis, California, USA; 9 Australian Centre for Ecogenomics, School of Chemistry and Molecular Biosciences, The University of Queensland, Brisbane, Australia

**Keywords:** strictly thermophilic, obligately chemolithoautotrophic, Gram-negative, aerobic, hydrogen-oxidizing, nonmotile, non sporeforming, rod shaped, *Aquificaceae*, *Aquificae*, GEBA

## Abstract

*Hydrogenobacter thermophilus* Kawasumi *et al*. 1984 is the type species of the genus *Hydrogenobacter*. *H. thermophilus* was the first obligate autotrophic organism reported among aerobic hydrogen-oxidizing bacteria. Strain TK-6^T^ is of interest because of the unusually efficient hydrogen-oxidizing ability of this strain, which results in a faster generation time compared to other autotrophs. It is also able to grow anaerobically using nitrate as an electron acceptor when molecular hydrogen is used as the energy source, and able to aerobically fix CO_2_ *via* the reductive tricarboxylic acid cycle. This is the fifth completed genome sequence in the family *Aquificaceae*, and the second genome sequence determined from a strain derived from the original isolate. Here we describe the features of this organism, together with the complete genome sequence and annotation. The 1,742,932 bp long genome with its 1,899 protein-coding and 49 RNA genes is a part of the *** G****enomic* *** E****ncyclopedia of* *** B****acteria and* *** A****rchaea * project.

## Introduction

Strain TK-6^T^ (= DSM 6534 = JCM 7687 = NBRC 102181) is the type strain of *Hydrogenobacter thermophilus*, which in turn is the type species of the genus *Hydrogenobacter* [1]. Currently, there are four validly published species in the genus *Hydrogenobacter*, one of which has subsequently been reclassified as *Hydrogenobaculum acidophilum*. Strain TK-6^T^ was previously isolated by Kawasumi in 1980 [2]. The genus name *Calderobacterium* Kryukov et al. 1984 is, based on page priority, a later heterotypic synonym of *Hydrogenobacter* Kawasumi et al. 1984 [3], because of similar genetic, phenotypic and biochemical properties between the type strains of *H. thermophilus* and *Calderobacterium hydrogenophilum*. Despite the relatively high degree of 16S rRNA gene sequence similarity between the two species, DNA-DNA hybridization [4] indicates that they may be considered to be different species within the genus *Hydrogenobacter* [3]. The genus name *Hydrogenobacter* is derived from the Latin words *hydrogenum*, meaning ‘that which produces water’ and *bacter*, referring to a rod that forms water when exposed to oxygen. The species epithet *thermophilus* derives from the Greek words *therme*, heat, and *philus*, loving, meaning a heat-loving organism. Strain TK-6^T^ was isolated from hot springs located on the Izu peninsula in Japan [1]. Some strains of *H. thermophilus* were also isolated from a geothermal spring in Tuscany, Italy [5,6]. Other strains similar to *H. thermophilus* have been isolated from different environments, including a saline hot spring in Japan for '*H. halophilus*' [7], and a volcanic area in Iceland for  *Hydrogenobacter* strain H-1 [8], strains T3, T13 and T171 [5]. Until 1985, *H. thermophilus* was the only obligate autotroph among all aerobic hydrogen-oxidizing bacteria reported so far [9,10]. The activities of enzymes such as NADH:ferredoxin reductase (EC 1.18.1.3) and NAD-reducing hydrogenase (EC 1.12.1.2) were studied extensively in strain TK-6^T^ [11]. Another genome sequence of a strain derived from the original isolate, presumably held in the lab of one of the co-authors, has been published recently without much metadata [12]. Here we present a summary classification and a set of features for *H. thermophilus* strain TK-6^T^, together with the description of the complete genomic sequencing and annotation.

## Classification and features

The 16S rRNA gene sequence of the strain TK-6^T^ (Z30214) shows the highest degree of sequence identity, 97%, to the type strain of *H. hydrogenophilus* [6]. Further analysis shows 96% 16S rRNA gene sequence identity with an uncultured *Aquificales* bacterium clone pKA (AF453505) from a near-neutral thermal spring in Kamchatka, Russia. The single genomic 16S rRNA sequence of *H. thermophilus* was compared with the most recent release of the Greengenes database [13] using NCBI BLAST under default values and the relative frequencies of taxa and keywords, weighted by BLAST scores, were determined. The five most frequent genera were *Hydrogenobacter* (52.4%), *Thermocrinis* (18.8%), *Aquifex* (10.3%), *Sulfurihydrogenibium* (6.2%) and *Hydrogenivirga* (5.7%). Regarding hits to sequences from other members of the genus, the average identity within HSPs (high-scoring segment pairs) was 96.1%, whereas the average coverage by HSPs was 93.5%. The species yielding the highest score was *H. hydrogenophilus*. The five most frequent keywords within the labels of environmental samples which yielded hits were 'hot' (6.5%), 'yellowstone' (5.8%), 'spring' (5.6%), 'national/park' (5.4%) and 'microbial' (3.9%). These keywords corroborate what is known from the ecology and physiology of strain TK-6^T^ [1,2]. The two most frequent keywords within the labels of environmental samples which yielded hits of a higher score than the highest scoring species were 'aquificales' (34.1%) and 'hot/spring' (32.9%).

**Figure 1:**
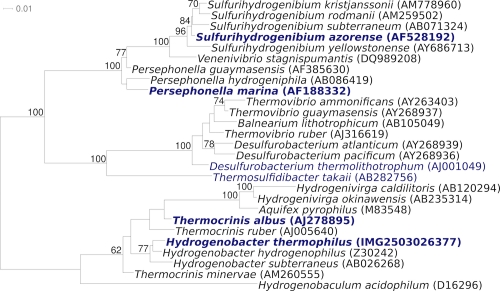
Phylogenetic tree highlighting the position of *H. thermophilus* TK-6^T^ relative to the type strains of the other species within the genus and to the type strains of the other genera within the family *Aquificaceae*. The trees were inferred from 1,423 aligned characters [14,15] of the 16S rRNA gene sequence under the maximum likelihood criterion [16] and rooted in accordance with the current taxonomy [17]. The branches are scaled in terms of the expected number of substitutions per site. Numbers above branches are support values from 1,000 bootstrap replicates [18] if larger than 60%. Lineages with type strain genome sequencing projects registered in GOLD [19] are shown in blue, published genomes in bold [12,20,21].

Figure 1 shows the phylogenetic neighborhood of *H. thermophilus* TK-6^T^ in a 16S rRNA based tree. The sequence of the single 16S rRNA gene in the genome differs by one nucleotide from the previously published 16S rRNA sequence (Z30214), which contains 31 ambiguous base calls.

**Figure 2:**
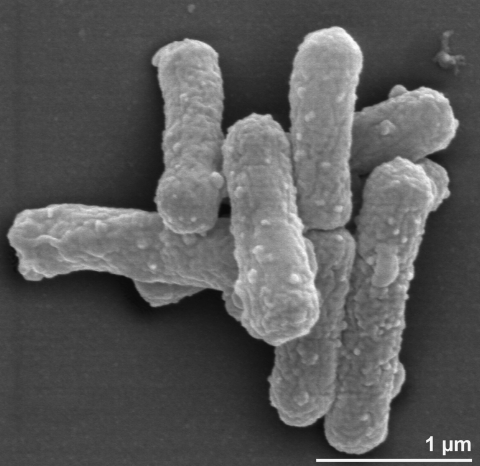
Scanning electron micrograph of *H. thermophilus* TK-6^T^

Cells of strain TK-6^T^ are Gram-negative, nonmotile straight rods of 0.3 to 0.5 µm by 2.0 to 3.0 µm occurring singly or in pairs [1] (Figure 2 and Table 1). Molecular oxygen is used as an electron acceptor for respiratory metabolism [1]. However, strain TK-6^T^ can grow anaerobically on nitrate as an electron acceptor when molecular hydrogen is used as an energy source [33]. Strain TK-6^T^ does not form colonies on agar plates, but does form colonies on plates solidified with GELRITE, a polysaccharide produced by *Pseudomonas* species [34]. The optimal temperature for autotrophic growth on H_2_-O_2_-CO_2_ was between 70ºC and 75°C, no growth being observed at 37°C or 80°C [1]. A neutral pH 7.2 was suitable for growth of the strain TK-6^T^ [1]. One important feature of the strain TK-6^T^ is a generation time that is faster by about 1h compared to other autotrophs, suggesting that this strain has an efficient hydrogen-oxidizing ability [35]. No spore formation was observed [1]. Strain TK-6^T^ assimilates carbon dioxide via the reductive tricarboxylic acid cycle [10,36,37]. This is also true when the strain TK-6^T^ grows anaerobically on nitrate [10]. Cytochromes *b* and *c* were found in strain TK-6^T^ [1]. Interestingly, cytochrome C_552_ of *H. thermophilus* TK-6^T^ is extremely thermostable and can restore its conformation even after being autoclaved for 10 minutes at 121ºC [30]. One of the denitrification enzymes of the strain TK-6^T^, cytochrome *cd*_1_ nitrite reductase has been isolated and analyzed [38]. Optimum temperature for the activity of this enzyme was found to range between 70ºC-75ºC [38]. Moreover, this enzyme was found to be of the heme *cd_1_*-type [33]. Ammonium and nitrate were utilized as nitrogen sources [1,33], but not urea and N_2_. Growth was inhibited by nitrite [1]. Nitrate reduction and peroxidase were positive, while urease was negative [1]. Strain TK-6^T^ could not utilize any of the following as sole sources of energy or carbon: glucose, fructose, galactose, maltose, sucrose, xylose, raffinose, L-rhamnose, D-mannose, D-trehalose, mannitol, starch, formate, acetate, propionate, pyruvate, succinate, malate, citrate, fumarate, maleate, glycolate, gluconate, DL-lactate, α-ketoglutarate, *p****-***hydroxybenzoate, DL-polyhydroxybutyrate, betaine, methanol, ethanol, methylamine, dimethylamine, trimethylamine, glycine, L-glutamate, L-aspartate, L-serine, L-leucine, L**-**valine, L-tryptophan, L-histidine, L-alanine, L-lysine, L-proline, L-arginine, nutrient broth, yeast extract-malt extract medium, and brain heart infusion [1]. Strain TK-6^T^ showed no growth under an atmosphere containing 90% CO, 5% CO_2_, and 5% O_2_ [1]. No heterotrophic growth was observed in the presence of glucose, fructose, pyruvate, citrate, α-ketoglutarate, succinate, fumarate, malate, acetate, and ethanol with and without yeast extract or carbon dioxide at different concentrations (0.02, 0.05, and 0.1% wt/vol) [1]. *H. thermophilus* TK-6^T^ was recently reported to grow on formate and formamide [39]. Malate dehydrogenase, isocitrate dehydrogenase and glucose-6-phosphate isomerase were also detected in the strain TK-6^T^ [1]. Enzymes of the reductive tricarboxylic acid cycle and some related enzymes in cell-free extracts of strain TK-6^T^ were detected and their specific activities were found to increase with the temperature, the enzymes being more active at 70°C, as compared to lower temperatures (50°C and 30°C) [10]. In *H. thermophilus*, ATP-dependent citrate cleavage is catalyzed by two enzymes, citryl-CoA synthetase and citryl-CoA lyase, which catalyze ATP-dependent formation of citryl-CoA from citrate and CoA and the subsequent cleavage of citryl-CoA into acetyl-CoA and oxaloacetate, respectively [40,41]. The biochemistry of key enzymes of the reductive tricarboxylic acid cycle, such as fumarate reductase, ATP citrate lyase, pyruvate:ferredoxin oxidoreductase  and 2-oxoglutarate:ferredoxin oxidoreductase, have been studied in some detail in strain TK-6^T^ [10,37,42]. Strain TK-6^T^ lacks some important enzyme activities in the central carbon metabolic pathways [43]. For example, activities of phosphofructokinase, pyruvate kinase, 6-phosphogluconate aldolase, which are key enzymes of the Embden-Meyerhof and the Entner-Doudoroff pathways, and activity of α-ketoglutarate dehydrogenase of the tricarboxylic acid cycle could not be detected in cell-free extracts of strain TK-6^T^ [43]. This is in accord with the findings from the genome sequencing where none of these genes were found in the genome. These metabolic deficits were considered to be partially responsible for the obligate autotrophy of the strain TK-6^T^ [44]. Activities of phosphoenolpyruvate synthetase and pyruvate carboxylase were also detected [10]. The reverse reactions (dehydrogenase reactions) of α-ketoglutarate synthase and pyruvate synthase could be detected by using methyl viologen as an electron acceptor [10]. Cloning experiments of the hydrogenase genes from the strain TK-6^T^ revealed that this strain has at least four clusters of hydrogenase genes [35]. Strain TK-6^T^ assimilates ammonium using glutamine synthetase (GS type I) [45]. Anisomycin, cycloheximide and emetine (100 µg/ml each) do not inhibit protein biosynthesis and therefore growth of strain TK-6^T^ [46]. But the inhibitors of  protein biosynthesis streptomycin, kanamycin, chloramphenicol, erythromycin, oleandomycin and virginiamycin were found to suppress growth of strain TK-6^T^ at concentrations below 20 µg/ml [46]. No growth was observed when cell wall synthesis inhibitors were used, (D-cycloserine, fosfomycin, cephalosporin C, penicillin G, oxacillin and ampicillin) at the concentration even below 20 µg/ml [46]. Strain TK-6^T^ could grow in the presence of monensin, lasalosid, valinomycin, nonactin and polymyxin B [46].

**Table 1:** Classification and general features of *H. thermophilus* TK-6^T^ according to the MIGS recommendations [22]

**MIGS ID**	**Property**	**Term**	**Evidence code**
	Current classification	Domain *Bacteria*	TAS [23]
		Phylum *Aquificae*	TAS [24,25]
		Class *Aquificae*	TAS [24,26]
		Order *Aquificales*	TAS [24,27,28]
		Family *Aquificaceae*	TAS [24,29]
		Genus *Hydrogenobacter*	TAS [1]
		Species *Hydrogenobacter thermophilus*	TAS [1]
		Type strain TK-6	TAS [1]
	Gram stain	negative	TAS [1]
	Cell shape	straight rods	TAS [1]
	Motility	non-motile	TAS [1]
	Sporulation	no	TAS [1]
	Temperature range	50°C–78°C	TAS [30]
	Optimum temperature	70°C-75°C	TAS [1]
	Salinity	not reported	NAS
MIGS-22	Oxygen requirement	aerobic	TAS [1]
	Carbon source	CO_2_	TAS [1]
	Energy source	H_2_, thiosulfate, obligate chemolithoautotrophic	TAS [1]
MIGS-6	Habitat	soil near hot spring	TAS [1]
MIGS-15	Biotic relationship	free living	NAS
MIGS-14	Pathogenicity	not reported	NAS
	Biosafety level	1	TAS [31]
	Isolation	hot spring	TAS [1]
MIGS-4	Geographic location	Izu peninsula, Japan	TAS [1,30]
MIGS-5	Sample collection time	1980 or before	TAS [1,2]
MIGS-4.1 MIGS-4.2	Latitude Longitude	approx. 34.9 138.9	NAS
MIGS-4.3	Depth	not reported	
MIGS-4.4	Altitude	not reported	

Evidence codes - IDA: Inferred from Direct Assay (first time in publication); TAS: Traceable Author Statement (i.e., a direct report exists in the literature); NAS: Non-traceable Author Statement (i.e., not directly observed for the living, isolated sample, but based on a generally accepted property for the species, or anecdotal evidence). These evidence codes are from of the Gene Ontology project [32]. If the evidence code is IDA, then the property was directly observed by one of the authors or an expert mentioned in the acknowledgements.

### Chemotaxonomy

The major cellular fatty acids found in strain TK-6^T^ were C_18:0_ and C_20:1_ [1,47]. These two fatty acids comprised about 80% of the total cellular fatty acids [1,47]. The minor components detected were C_16:0_, C_16:1_ and C_18:1_. C14:0 acids (indicative of the presence of a lipopolysaccharide) and a C_21:0_ cyclopropane acid, representing less than 10% of the total cellular fatty acids [1,47]. The detailed fatty acid composition of the strain TK-6T is available in [27] and [47]. The main respiratory lipoquinone is an unusual sulfur-containing quinone, a 2-methylthio-3-VI, VII-tetrahydroheptaprenyl-1,4-naphthoquinone (i.e., methionaquinone 7, MTK-7) [48,49]. Strain TK-6T contains glycerol-ether basedlipids, as well as acyl glycerides [47]. It should be noted that the ether lipids are not of the type found in members of the *Archaea*, since the side chains are alkyl straight chain and not isoprenoid. The presence of glycerol monoethers (GME) (1.2 µ mol/g dwt) is a characteristic feature of the strain TK-6T, the main one being GME-18:0 (82.7% wt) [27,47]. GME-20:1 (11.1% wt), GME-20:0 (3.5 wt), and GME-18:1 (2.7% wt) were also detected in strain TK-6T [27,47]. No glycerol diether (GDE) was detected [27,47]. Investigations of the polar lipids have shown that they comprise phosphatidylglycerol, phosphatidylinositol, phosphatidylaminopentantetrol and a small amount of an unidentified phospholipid. The sum of these chemotaxomonic features appears to be characteristic of members of the genus *Hydrogenobacter*, with features such as the presence of methionaquinone, a polar lipid pattern containing phosphatidylglycerol, phosphatidylinositol and phosphatidylaminopentantetrol and the presence of C_18:0_ and C_20:1_ fatty acids being taxonomic and evolutionary markers for at least members of the genera *Hydrogenobacter*, *Hydrogenobaculum*, *Aquifex* and *Thermoncrinis*. This has been discussed in a previous SIGS paper [50].

## Genome sequencing and annotation

### Genome project history

This organism was selected for sequencing on the basis of its phylogenetic position [51], and is part of the *** G****enomic* *** E****ncyclopedia of* *** B****acteria and* *** A****rchaea * project [52]. The genome project is deposited in the Genome On Line Database [19] and the complete genome sequence is deposited in GenBank. Sequencing, finishing and annotation were performed by the DOE Joint Genome Institute (JGI). A summary of the project information is shown in Table 2.

**Table 2:** Genome sequencing project information

**MIGS ID**	**Property**	**Term**
MIGS-31	Finishing quality	Finished
MIGS-28	Libraries used	One 454 pyrosequence standard library, one 454 PE (20kb insert size) and one Illumina standard library
MIGS-29	Sequencing platforms	454 GS FLX Titanium, Illumina GAii
MIGS-31.2	Sequencing coverage	82.1× pyrosequence, 264.4 × Illumina
MIGS-30	Assemblers	Newbler version 2.3-PreRelease-10-21-2009-gcc-4.1.2, phrap
MIGS-32	Gene calling method	Prodigal 1.4, GenePRIMP
	INSDC ID	CP002221
	Genbank Date of Release	October 15, 2010
	GOLD ID	Gc01411
	NCBI project ID	41547
	Database: IMG-GEBA	2502957034
MIGS-13	Source material identifier	DSM 6534
	Project relevance	Tree of Life, GEBA

### Growth conditions and DNA isolation

*H. thermophilus* TK-6^T^, DSM 6534, was grown in DSMZ medium 533 (Thermophilic hydrogen bacteria medium) [53] with 5% oxygen at 72°C. DNA was isolated from 0.5-1 g of cell paste using Qiagen Genomic 500 DNA Kit (Qiagen, Hilden, Germany) following the standard protocol as recommended by the manufacturer. DNA is available through the DNA Bank Network [54].

### Genome sequencing and assembly

The genome was sequenced using a combination of Illumina and 454 sequencing platforms. All general aspects of library construction and sequencing can be found at the JGI website [55]. Pyrosequencing reads were assembled using the Newbler assembler version 2.3-PreRelease-10-21-2009-gcc-4.1.2-threads (Roche). The initial Newbler assembly consisted of 19 contigs in one scaffold which was converted into a phrap assembly by making fake reads from the consensus, collecting the read pairs in the 454 paired end library. Illumina GAii sequencing data (449.5 Mb) was assembled with Velvet [56] and the consensus sequences were shredded into 1.5 kb overlapped fake reads and assembled together with the 454 data. The 454 draft assembly was based on 143.2 MB 454 draft data and all of the 454 paired end data. Newbler parameters are -consed -a 50 -l 350 -g -m -ml 20. The Phred/Phrap/Consed software package [57] was used for sequence assembly and quality assessment in the subsequent finishing process. After the shotgun stage, reads were assembled with parallel phrap (High Performance Software, LLC). Possible mis-assemblies were corrected with gapResolution [55], Dupfinisher, or sequencing cloned bridging PCR fragments with subcloning or transposon bombing (Epicentre Biotechnologies, Madison, WI) [58]. Gaps between contigs were closed by editing in Consed, by PCR and by Bubble PCR primer walks (J.-F.Chang, unpublished). A total of 24 additional Sanger reactions were necessary to close gaps and to raise the quality of the finished sequence. Illumina reads were also used to correct potential base errors and increase consensus quality using a software Polisher developed at JGI [59]. The error rate of the completed genome sequence is less than 1 in 100,000. Together, the combination of the Illumina and 454 sequencing platforms provided 346.5 × coverage of the genome. Final assembly contains 454,097 pyrosequence and 12,484,847 Illumina reads.

### Genome annotation

Genes were identified using Prodigal [60] as part of the Oak Ridge National Laboratory genome annotation pipeline, followed by a round of manual curation using the JGI GenePRIMP pipeline [61]. The predicted CDSs were translated and used to search the National Center for Biotechnology Information (NCBI) nonredundant database, UniProt, TIGRFam, Pfam, PRIAM, KEGG, COG, and InterPro databases. Additional gene prediction analysis and functional annotation was performed within the Integrated Microbial Genomes - Expert Review (IMG-ER) platform [62].

## Genome properties

**Figure 3:**
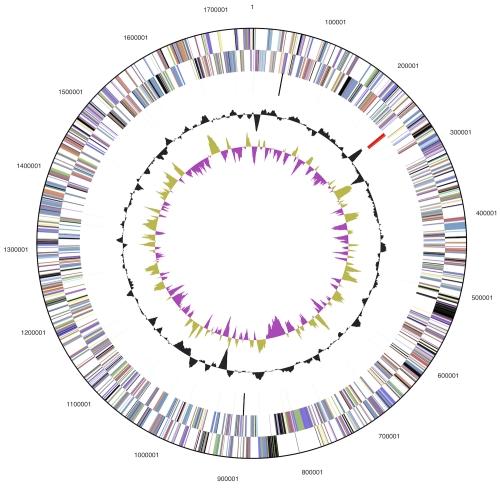
Graphical circular map of the genome. From outside to the center: Genes on forward strand (color by COG categories), Genes on reverse strand (color by COG categories), RNA genes (tRNAs green, rRNAs red, other RNAs black), GC content, GC skew.

The genome consists of a 1,742,932 bp long chromosome with a 44.0% G+C content (Table 3 and Figure 3). Of the 1,948 genes predicted, 1,899 were protein-coding genes, and 49 RNAs; thirty pseudogenes were also identified. The majority of the protein-coding genes (97.5%) were assigned with a putative function while the remaining ones were annotated as hypothetical proteins. The distribution of genes into COGs functional categories is presented in Table 4.

**Table 3:** Genome Statistics

**Attribute**	**Value**	**% of Total**
Genome size (bp)	1,742,932	100.00%
DNA coding region (bp)	1,666,175	95.60%
DNA G+C content (bp)	766,905	44.00%
Number of replicons	1	
Extrachromosomal elements	0	
Total genes	1,948	100.00%
RNA genes	49	2.52%
rRNA operons	1	
Protein-coding genes	1,899	97.48%
Pseudo genes	30	1.54%
Genes with function prediction	1,361	69.87%
Genes in paralog clusters	183	9.39%
Genes assigned to COGs	1,441	73.97%
Genes assigned Pfam domains	1,501	77.05%
Genes with signal peptides	287	14.73%
Genes with transmembrane helices	381	19.56%
CRISPR repeats	1	

**Table 4:** Number of genes associated with the general COG functional categories

**Code**	**value**	**%age**	**Description**
J	134	8.6	Translation, ribosomal structure and biogenesis
A	0	0.0	RNA processing and modification
K	52	3.3	Transcription
L	85	5.4	Replication, recombination and repair
B	2	0.1	Chromatin structure and dynamics
D	19	1.3	Cell cycle control, cell division, chromosome partitioning
Y	0	0.0	Nuclear structure
V	21	1.3	Defense mechanisms
T	53	3.4	Signal transduction mechanisms
M	128	8.2	Cell wall/membrane/envelope biogenesis
N	23	1.5	Cell motility
Z	1	0.0	Cytoskeleton
W	0	0.0	Extracellular structures
U	56	3.6	Intracellular trafficking and secretion, and vesicular transport
O	74	4.7	Posttranslational modification, protein turnover, chaperones
C	182	11.6	Energy production and conversion
G	58	3.7	Carbohydrate transport and metabolism
E	118	7.5	Amino acid transport and metabolism
F	52	3.3	Nucleotide transport and metabolism
H	107	6.8	Coenzyme transport and metabolism
I	43	2.7	Lipid transport and metabolism
P	78	5.0	Inorganic ion transport and metabolism
Q	15	1.0	Secondary metabolites biosynthesis, transport and catabolism
R	167	10.7	General function prediction only
S	100	6.4	Function unknown
-	507	26.3	Not in COGs

## Insights into the genome

While the sequencing of the genome described in this paper was underway, Arai *et al.* from University of Tokyo published the first version of the *H. thermophilus* TK-6^T^ genome [19, AP011112]. We take the opportunity to compare the two completed genome sequences, because the history of the two strains designated TK-6^T^ might differ since the original isolation of the strain by Kawasumu *et al*. [1], more than a 25 years ago. The first of the two genomes was published by a team of researchers located at the same place where the strain was originally analyzed, with Yasuo Igarashi participating in both, the original description of the strain and the genome analysis. According to personal information by Dr. Arai Hiroyuki (lead author in [19]), the genome was sequenced from clone and fosmid libraries generated by a strain subcultured in the lab since the time of the initial isolation. A fresh culture of the strain from JCM was used for final gap filling and error checking. The DSM 6534 version of the genome was generated from cryopreserved material, which DSMZ received in 1991 from Tohru Kodama of University of Tokyo, and the strain was preserved by storage in liquid nitrogen since it was accessed.

A comparison of the two TK-6^T^ genomes using the genome-to-genome-distance calculation [63-65] in conjunction with NCBI-BLASTN yielded a distance of 0.0001 with formula 1, 0.0100 with formula 2 and 0.0101 with formula 3. That is, 99.99% of the total genome length was covered by HSPs, 99.0% of the positions within the HSPs held identical bases, and 98.99% of the total genome length corresponded to such identical base pairs within HSPs. The synteny of the two TK-6^T^ genome sequences based on a DNA blot was confirmed (data not shown), whereas Table 5 provides a comparison of the basic genome statistics.

**Table 5:** Comparison of Genome Statistics

**Attribute**	DSM 6534	U of Tokyo	difference
Genome size (bp)	1,742,932	1,744,135	+1,203
DNA coding region (bp)	1,666,175	1,669,712	+3,537
DNA G+C content (bp)	766,905	766,984	+79
Number of replicons	1	1	1
Extrachromosomal elements	0	0	0
Total genes	1,948	1,941	-7
RNA genes	49	48	-1
rRNA operons	1	1	1
Protein-coding genes	1,899	1,893	-6
Pseudo genes	30	0	-30
Genes with function prediction	1,361	1,349	-12
Genes in paralog clusters	183	175	-8
Genes assigned to COGs	1,441	1,430	-11
Genes assigned Pfam domains	1,501	1,489	-12
Genes with signal peptides	287	528	+241
Genes with transmembrane helices	381	385	+4
CRISPR repeats	1	2	+1

The Japanese strain has 1,868 (out of 1,893) protein coding genes identical to the DSMZ strain which is 98.7% of the genome. This means there are 25 genes in the Japanese strain that are not in the DSMZ strain, all except L34P are hypothetical genes. L34P is however present in the version of the genome as presented in this paper, but was missed from the ORF calling/annotation. We also identified 24 genes in the genome sequenced from the DSMZ strain that were missing in the Arai *et al.* strain. Also most of these were again hypothetical genes. The abundance profiles for both genomes were almost identical, with glycosyltransferase (COG0438) being the most frequent gene in both versions (eleven copies), followed by seven copies of an outer membrane protein (COG1538), each. The DSM 6534 genome contains seven copies of transposase IS605 OrfB (COG0675), whereas Tokyo contains five copies of it.

The DSM 6534 version of the genome also contains more copies of cation transport ATPase (COG0474, 4 *vs*. 2), nitrogenase molybdenum-iron protein, alpha and beta chains (COG2710, 4 *vs*. 2), acetyl/propionyl-CoA carboxylase, alpha subunit (COG4779, 4 *vs*. 3), Fe-S oxidoreductases (GCO0474, 3 *vs*. 2), catabolite gene activator and regulatory subunit of cAMP-dependent protein kinases (COG0664, 3 *vs*. 2), cation transport ATPase (COG2217, 3 *vs*. 2), DNA modification methylase (COG0862, 2 *vs*. 1), hemolysins and related proteins containing CBS domains (COG1253, 2 *vs*. 1). Phosphoketolase (COG3957), an uncharacterized MobA-related protein (COG2068) and an uncharacterized conserved protein (COG4121) were identified in one copy, each, in the DSM 6534 genome, but absent in the U Tokyo version. The U Tokyo version contains more copies of selenocysteine-containing anaerobic dehydrogenases, (COG0243, 5 *vs*. 1), as well as,1-acyl-sn-glycerol-3-phosphate acyltransferase (COG02043) and K+-transporting ATPase, A chain (COG2060, 2 *vs*. 1, each)
